# The use of a benign fast-growing cyanobacterial species to control microcystin synthesis from *Microcystis aeruginosa*

**DOI:** 10.3389/fmicb.2024.1461119

**Published:** 2024-12-05

**Authors:** Hakyung Lee, Vincent Xu, Jinjin Diao, Runyu Zhao, Moshan Chen, Tae Seok Moon, Haijun Liu, Kimberly M. Parker, Young-Shin Jun, Yinjie J. Tang

**Affiliations:** ^1^Department of Energy, Environmental and Chemical Engineering, Washington University in St. Louis, St. Louis, MO, United States; ^2^Department of Biology, Saint Louis University, St. Louis, MO, United States

**Keywords:** coculture, DCMU, harmful algal blooms, *Microcystis aeruginosa*, *Synechococcus elongatus*

## Abstract

**Introduction:**

*Microcystis aeruginosa* (*M. aeruginosa*), one of the most abundant blue-green algae in aquatic environments, produces microcystin by causing harmful algal blooms (HABs). This study investigated the combined effects of nutrients and competition among cyanobacterial subpopulations on the synthesis of microcystin-LR.

**Methods:**

Under varying nitrogen and phosphorus concentrations, cyanobacterial coculture, and the presence of algicidal DCMU, the growth was monitored by optical density analysis or microscopic counting, and the microcystin production was analyzed using high-performance liquid chromatography-UV. Furthermore, growth and toxin production were predicted using a kinetic model.

**Results and discussion:**

First, coculture with the fast-growing cyanobacterium *Synechococcus elongatus* UTEX 2973 (*S. elongatus*) reduced *M. aeruginosa* biomass and microcystin production at 30°C. Under high nitrogen and low phosphorus conditions, *S. elongatus* was most effective, limiting *M. aeruginosa* growth and toxin synthesis by up to 94.7% and 92.4%, respectively. Second, this biological strategy became less effective at 23°C, where *S. elongatus* grew more slowly. Third, the photosynthesis inhibitor DCMU (3-(3,4-dichlorophenyl)-1,1-dimethylurea) inhibited *M. aeruginosa* growth (at 0.1 mg/L) and microcystin production (at 0.02 mg/L). DCMU was also effective in controlling microcystin production in *S. elongatus*–*M. aeruginosa* cocultures. Based on the experimental results, a multi-substrate, multi-species kinetic model was built to describe coculture growth and population interactions.

**Conclusion:**

Microcystin from representative toxin-producing *M. aeruginosa* can be controlled by coculturing fast-growing benign cyanobacteria, which can be made even more efficient if appropriate algicide is applied. This study improved the understanding of the biological control of microcystin production under complex environmental conditions.

## 1 Introduction

Harmful algal blooms (HABs) are a global water problem, continuously making headlines, such as the cases of the Gulf of Mexico (Ulloa et al., 2017), the North American Great Lakes (Carmichael and Boyer, 2016), and Lake Taihu in China (Michalak et al., 2013; Ho and Michalak, 2015). HABs are not only visually undesirable but also produce toxins that are harmful to human health and ecosystems (Carmichael and Boyer, 2016). *Microcystis aeruginosa* (*M. aeruginosa*) is one of the most abundant and diverse cyanobacteria (blue–green algae) that are classified as either toxigenic or non-toxigenic (Dick et al., 2021). Toxigenic strains produce a class of hepatotoxins called microcystins, capable of damaging the organisms (Ouellette et al., 2006; Carmichael and Gorham, 1981). Its *mcy* gene regulates the congener composition of microcystin under various environmental factors, and microcystin-LR (named for its leucine and arginine contents) is the most common congener (Bashir et al., 2023; Yancey et al., 2022).

Because of the sublethal effects of microcystin, especially on the living organism’s liver in both acute and chronic ways, the World Health Organization has issued the microcystin guideline value of 1 μg/L for drinking water and 24 μg/L for recreational water (Chorus and Welker, 2021). Microcystin-related bloom control strategies have been actively explored to respond to global microcystin regulations (Ma et al., 2014; Graham et al., 2004; Jiang et al., 2008). Physicochemical treatment of harmful algal blooms, such as coagulation with metal salts, hydrodynamic cavitation, and plasma, has been developed (Gonzalez-Torres et al., 2014; Li P. et al., 2015; Xu et al., 2022). To overcome the limitations of physical and chemical solutions such as sustainability and scalability, biological control methods have been highlighted (Choi et al., 2005). Studies have successfully examined the coculture of *M. aeruginosa* with other species (e.g., *Bacillus*, *Brevibacillus*, and *Chryseobacterium*) under different environmental conditions such as temperature and light intensity (Li Z. et al., 2015b; Zhang et al., 2021; Zhang et al., 2019; Yang et al., 2015; Li et al., 2020).

Nitrogen-and phosphorus-related eutrophication is the primary cause of harmful algal blooms (Watson et al., 2016; Granéli et al., 2008). While studies have examined the coculture strategy to manage *M. aeruginosa* blooms in various physical conditions, the coculture strategy considering nutrient conditions remains unclear. Therefore, this study came up with the idea of coculturing other species that can uptake the nutrients that *M. aeruginosa* is supposed to eat. *Synechococcus elongatus* UTEX 2973 (*S. elongatus*) is the test candidate, considering its fast growth and environment-friendliness. Because of its fast growth rate, *Synechococcus elongatus* has been used to capture CO_2_ and produce biochemicals and nutraceuticals (Yu et al., 2015). Unlike parasite-based biological controls that may cause unintended environmental impacts, *S. elongatus* provides animal food and biomanufacturing feedstock (Song et al., 2016; Lin et al., 2020).

This study aimed to test the growth and toxin production behavior of *M. aeruginosa* in the presence of *S. elongatus*. Our core hypothesis is that coculturing non-toxic *S. elongatus* will significantly control *M. aeruginosa* containing HAB by the competition of nitrogen (N) and phosphorus (P). We were curious about how the coculture with *S. elongatus* affects *M. aeruginosa* growth and microcystin synthesis under various nutrients and whether this approach and other chemical bloom control strategies synergize. Therefore, we explored the relationship of nitrogen (N) and phosphorus (P) with algal biomass and microcystin generation in the coculture system. Previous studies have shown that N depletion decreases total microcystin and biomass concentrations (Horst et al., 2014). However, P depletion does not always alleviate the cyanotoxin generation (Briand et al., 2012; Oh et al., 2000). The trend in *S. elongatus* coculture needs to be clarified. Assuming that the coculture controls the *M. aeruginosa* growth, the effectiveness of the synergistic control using chemical algicides (such as photosynthesis inhibitors) that are often used to treat HABs was our interest expecting the improvement of the bloom control efficiency (Liu et al., 2023; Greenfield et al., 2014; Kirilovsky et al., 1994; Yilimulati et al., 2022; Küpper et al., 1996). Among various algicides, we selected 3-(3,4-dichlorophenyl)-1,1-dimethylurea (DCMU). DCMU was initially introduced as an herbicide to increase crop productivity. The mode of action blocks the electron transfer by binding to the plastoquinone binding site of photosystem II (Giacomazzi and Cochet, 2004). Knowing the efficiency of DCMU controlling photosynthesis, it has been actively studied as a conventional algicide to control harmful algal bloom-forming photosynthetic microorganisms, and it is affordable and easy to access (Huang et al., 2024; Zhou et al., 2013; Zhang et al., 2022). The conservative target machinery of photosynthesis does not significantly affect organisms that do not have photosystem (Liu, 2014; Tayor, 1997). The lethal dosage of DCMU for non-photosynthetic living organisms such as humans and aquatic animals is high, requiring higher than-environmental concentration (Krieger, 2001). In the study, we carefully determined the DCMU dosage and aimed to apply the minimum necessary amount, considering the non-selectivity of DCMU that may affect other phytoplankton.

Based on the experimental findings, we built a Monod-based kinetic model to simulate the effects of varying nutrient levels and biological competition conditions on *M. aeruginosa* growth and microcystin production. This study will obtain new concepts for the biological control of HABs.

## 2 Materials and methods

### 2.1 Strains and algicide

Cyanobacterial species were purchased from the University of Texas at Austin, Culture Collection of Algae (UTEX, Austin). This study used *M. aeruginosa* UTEX LB 2385 as a model strain, showing similar growth behavior to other microcystin-generating *M. aeruginosa* strains (Chen et al., 2019; Pimentel and Giani, 2014). For the seed culture preparation, *M. aeruginosa* was grown with Bold 3 N medium, and *S. elongatus* UTEX 2973 was grown with the BG 11 medium (Chen et al., 2020). The medium composition is summarized in Appendix A. 3-(3,4-Dichlorophenyl)-1,1-dimethylurea (DCMU) was purchased from Sigma-Aldrich (catalog number: D2425). The concentrated DCMU stock was prepared in acetone and diluted to a working concentration during the sample preparation. The acetone concentration in the final sample was less than 1%.

### 2.2 Cyanobacteria cultivation

N- and P-modified Bold 3 N media were used for algal growth. The media contained three different N and P concentrations (N: 1.235, 12.35, and 123.5 mg/L; P: 0.53, 5.33, and 53.25 mg/L) (Cox and Bold, 1966). We defined these concentrations as the low, medium, and high N or P conditions. The medium N and P conditions correspond to the environmental aquatic nutrient level. All cultures (50 mL working volume) were performed in 250-ml shake flasks at 150 rpm under 23 or 30°C, continuous light (200 μmol/photons/m^2^/s), and atmospheric CO_2_ conditions. To prepare the inoculum, the seed culture was grown to the exponential phase (OD_730_ ~ 1) in the shaking flask. Then biomass was centrifuged at 10,000 rcf (relative centrifugal force) for 10 min at room temperature. The biomass pellets were resuspended in 0.9% NaCl solution; the resuspended cultures were used for inoculation at 5% v/v (seed culture volume was 5% of the total sample volume for each inoculation). The ratio of inoculated species for coculture with *S. elongatus* was 1:1 (total inoculant was 5% v/v). All experiments were performed in triplicate. Cell growth was monitored by OD_730_ or microscope imaging. To count cell numbers, *M. aeruginosa* (round shape) and *S. elongatus* (rod shape) were measured using a C-Chip hemocytometer (INCYTO, South Korea, Catalog number: DHC-N01) and an optical microscope (Zeiss Axio Observer ZI Inverted Microscope, Zeiss).

### 2.3 Microcystin, N, and P analyses

Microcystin analysis followed reported protocols (the detailed procedure is in Appendix B; Shoemaker et al., 2015; Zervou et al., 2017). In brief, extracted microcystin samples were analyzed using an Agilent 1,260 Infinity High-Performance Liquid Chromatography-UV (HPLC-UV) instrument equipped with an Agilent Eclipse Plus C18 column (3.0 mm × 150 mm, 3.5 μm). The aqueous phase was formic acid/water (0.1/99.9, v/v), and the organic phase was acetonitrile/water (99/1, v/v). The flow rate was 0.5 mL/min, and the injection volume was 10 μL. Gradient elution started at 90% aqueous phase for 7 min, was adjusted to 65% at 7.1 min, and was held constant until 10 min had elapsed, followed by a linear decrease to 45% at 16 min. Then, it was adjusted to 90% at 16.1 min. The retention time of MC-LR was 15 min. The UV absorbance wavelength was 239 nm (Matthiensen et al., 2000). The limit of detection of MC-LR was 0.1 mg/L, determined from standards prepared using microcystin-LR solution (Sigma-Aldrich, catalog number 33893). The analysis was performed in triplicate. The microcystin concentration was converted to the cell quota by dividing the total number of cells in each test condition counted during the growth test. In addition, NO_3_^−^ (N) and PO_4_^3−^ (P) were quantified by anion chromatography using an IonPac AS18 (Dionex Integrion HPIC, WI, United States).

### 2.4 RT-qPCR analysis

*Microcystis aeruginosa* samples at the late growth phase were collected, and their expressions of *mcy*B and *mcy*D genes were examined to quantify microcystin-producing activities (Dittmann et al., 2001). Cells were grown under high and low P conditions with a high N supplement to reveal the effect of P limitation. Biomass was spun down, and the cell pellet was quenched in liquid N_2_. RNA was extracted using the RNA Plant Mini Kit (QIAGEN, Germany). Cell lysis during the extraction was conducted using a BeadBug™ microtube homogenizer inside a BeadBug prefilled tube (BeadBug, NJ). After removing gDNA, cDNA reverse transcription was performed using a PrimeScript™ RT Reagent Kit (Takara, Japan). Then, we added the cDNA, 0.3 μL of each primer with the concentration of 10 μM (forward and reverse, Appendix C, purchased from Integrated DNA Technologies, IA.), and 5 μL of PowerUp™ SYBR™ Green Master Mix (Applied Biosystems™, MA) in a PCR tube, which was then filled with sterile water to make a 10 μL volume. The gene expressions were quantified using a CFX Connect RT-PCR Detection System (Bio-Rad, CA). The sample was heated for 30 s at 95°C, and the reaction was conducted for 40 cycles of 5 s at 95°C, 10 s at 55°C, and 20 s at 72°C. After that, the melting curve stability was determined. The temperature increased at a rate of 0.5°C/s from 65°C to 95°C. The 16S ribosomal RNA gene was the internal control. 2^-ΔΔCt^ fold change of gene expression was determined.

### 2.5 Statistical analysis

Statistical analysis was conducted using GraphPad Prism 10. Triplicate data were used to calculate the mean ± standard error (SE) for growth, nutrient consumption, and microcystin concentration. The parameter difference was tested using a one-way analysis of variance (ANOVA) or *t*-test with a significance level of *p* < 0.05. The Pearson coefficient using R (version 3.3.0) determined a correlation between nitrogen concentration and microbial growth.

### 2.6 Model development and simulations

A Monod-based model for algal growth and toxin production was used (Xiao et al., 2021; Martínez et al., 1997; Aslan and Kapdan, 2006; Spijkerman et al., 2011):


(1) $$\begin{array}{c} \frac{dX_{i}}{dt}=\mu_{\max,i}\frac{N}{K_{N_{i}}+N}\frac{P}{K_{P_{i}}+P}\frac{S_{CO_{2}}}{{K_{CO_{2}}}_{i}+S_{CO_{2}}}\frac{I}{{K_{L}}_{i}+I}X_{i}\\ -{k_{d}}_{i}X_{i}\;\left(i=A,B\right)\text{.} \end{array}$$

*A* and *B* were the biomass X for *M. aeruginosa* and *S. elongatus*, respectively. *N*, *P*, *Sco_2_*, and *I* were N, P, and CO_2_ concentrations and light intensity, respectively. μ_max_ and k_d_ were the specific rate constants for cell growth and death, respectively. Based on the Beer–Lambert law, a light density function included variables of shading from total biomass (*X_A_ + X_B_*) and light absorption (*A*) of the photobioreactor (Martínez et al., 1997). The microcystin production *MC* was related to biomass growth. An empirical term α = β (N/P)^n^ described the toxin accumulation as a function of N:P ratios:


(2) $$I=\left(\frac{I_{0}}{A\;.\;\left(X_{A}+X_{B}\right)}\right)\left(1-\exp\left(-A\;.\;\left(X_{A}+X_{B}\right)\right)\right)\text{.}$$

(3) $$\frac{dMC}{dt}=\alpha \mu_{\text{max}}\frac{N}{K_{N_{A}}+N}\frac{P}{K_{P_{A}}+P}\frac{S_{CO_{2}}}{K_{CO_{2_{A}}}+S_{CO_{2}}}\frac{I}{K_{L_{A}}+I}X\text{.}$$

N and P consumptions were predicted based on the yield coefficients (Y_X/N_ and Y_X/P_).


(4) $$\frac{dN}{dt}=-\sum_{i=A,B}Y_{\frac{X}{N}}\mu_{\max,i}\frac{N}{K_{N_{i}}+N}\frac{P}{K_{P_{i}}+P}\frac{S_{CO_{2}}}{{K_{CO_{2}}}_{i}+S_{CO_{2}}}\frac{I}{{K_{L}}_{i}+I}X_{i}$$

(5) $$\frac{dP}{dt}=-\sum_{i=A,B}Y_{\frac{X}{P}}\mu_{\max,i}\frac{N}{K_{N_{i}}+N}\frac{P}{K_{P_{i}}+P}\frac{S_{CO_{2}}}{{K_{CO_{2}}}_{i}+S_{CO_{2}}}\frac{I}{{K_{L}}_{i}+I}X_{i}$$

For CO_2_ consumption, the air–water mass transfer was considered.


(6) $$\begin{array}{c} \frac{dS_{CO_{2}}}{dt}=K_{L_{a}}\left({S_{CO_{2}}}^{*}-S_{CO_{2}}\right)\\ -\sum_{i=A,B}Y_{\frac{X}{C}}\mu_{\max,i}\frac{N}{K_{N_{i}}+N}\frac{P}{K_{P_{i}}+P}\frac{S_{CO_{2}}}{{K_{CO_{2}}}_{i}+S_{CO_{2}}}\frac{I}{{K_{L}}_{i}+I}X_{i} \end{array}$$

Baseline parameters for a sensitivity analysis under various stress conditions were selected and estimated based on the literature and the experiments listed in Appendix D. The MATLAB code with *ode45* functions is provided in Appendix E.

## 3 Results

### 3.1 Effects of N and P on *Microcystis aeruginosa* monoculture growth and microcystin production

**Figure 1:**
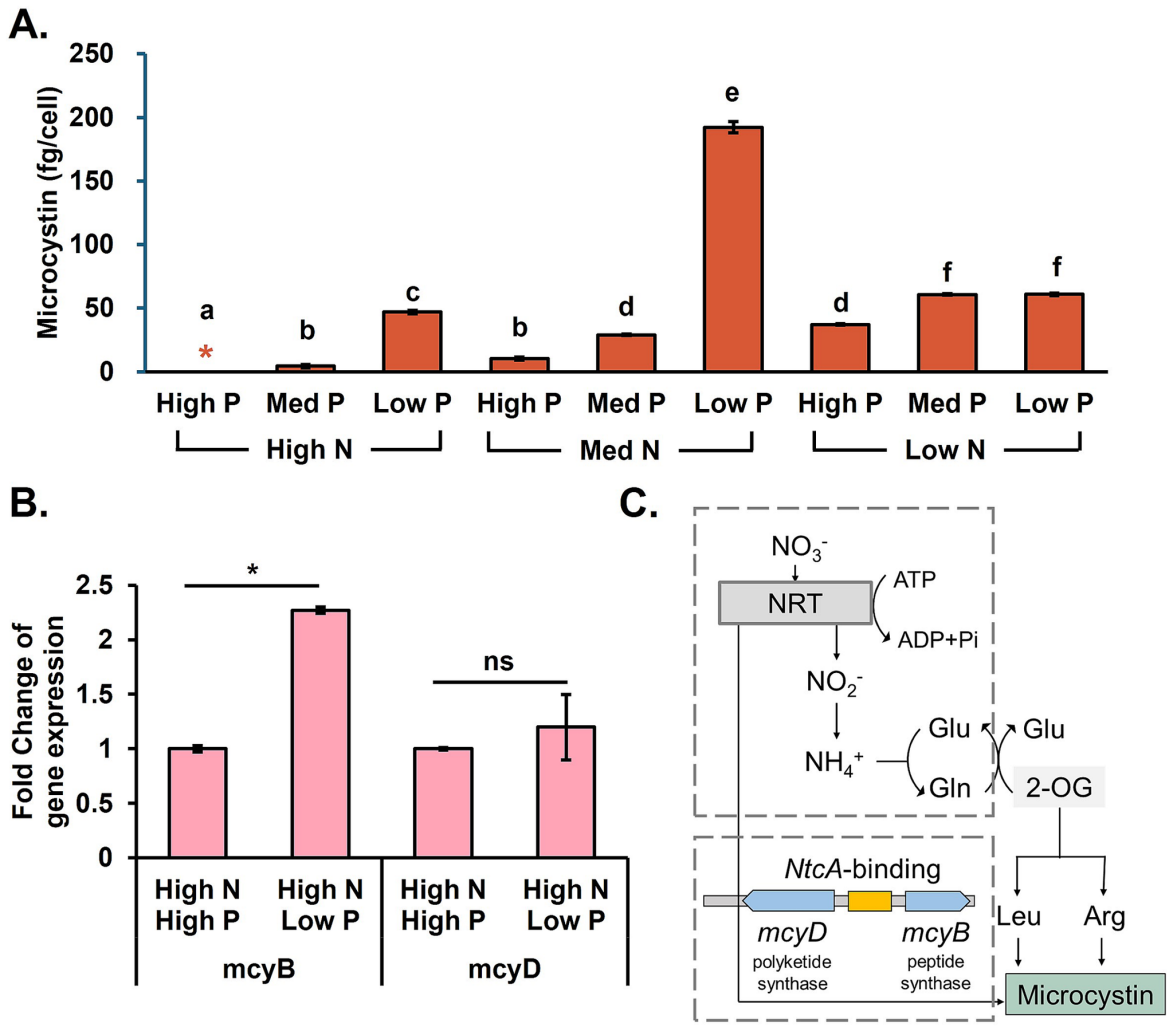
Microcystin production in *M. aeruginosa* monoculture. **(A)** Final microcystin production, **(B)** microcystin-producing gene expression profile under different P concentrations, and **(C)** microcystin synthesis pathways. Different letters signify distinguishable differences observed across different nutrient conditions (ANOVA, *p* < 0.05). Black asterisks indicate a significant difference compared to the same N and higher P conditions, and ns stands for no significance (*t*-test, *p* < 0.05).

Microbial growth and microcystin production tests were performed in the *M. aeruginosa* monoculture for the control study. The cell density in the stationary phase showed that *M. aeruginosa* biomass production had a strong positive correlation with N concentration, with a Pearson coefficient of 0.78 (*p* < 0.01; Appendix F). As expected, the growth, shown as the cell density, was highest when N and P were sufficient. However, a higher final *M. aeruginosa* population did not necessarily lead to increased microcystin (Figure 1). On the other hand, when N was deficient in the medium, both biomass production and microcystin production were limited. *M. aeruginosa* growth rate was 84.7% lower when P was deficient in the medium. Low growth did not correlate with less microcystin. For instance, highest microcystin cell quota was observed under low P but medium N concentrations. High N and low P conditions favored microcystin production (Oh et al., 2000; Briand et al., 2012). This observation was consistent with our RT-qPCR results that showed higher microcystin-producing gene (*mcyB*) expression induced by P limitation (Figure 1B; Lee, 2024).

### 3.2 Effect of *Synechococcus elongatus* on *Microcystis aeruginosa* growth and microcystin production

**Figure 2:**
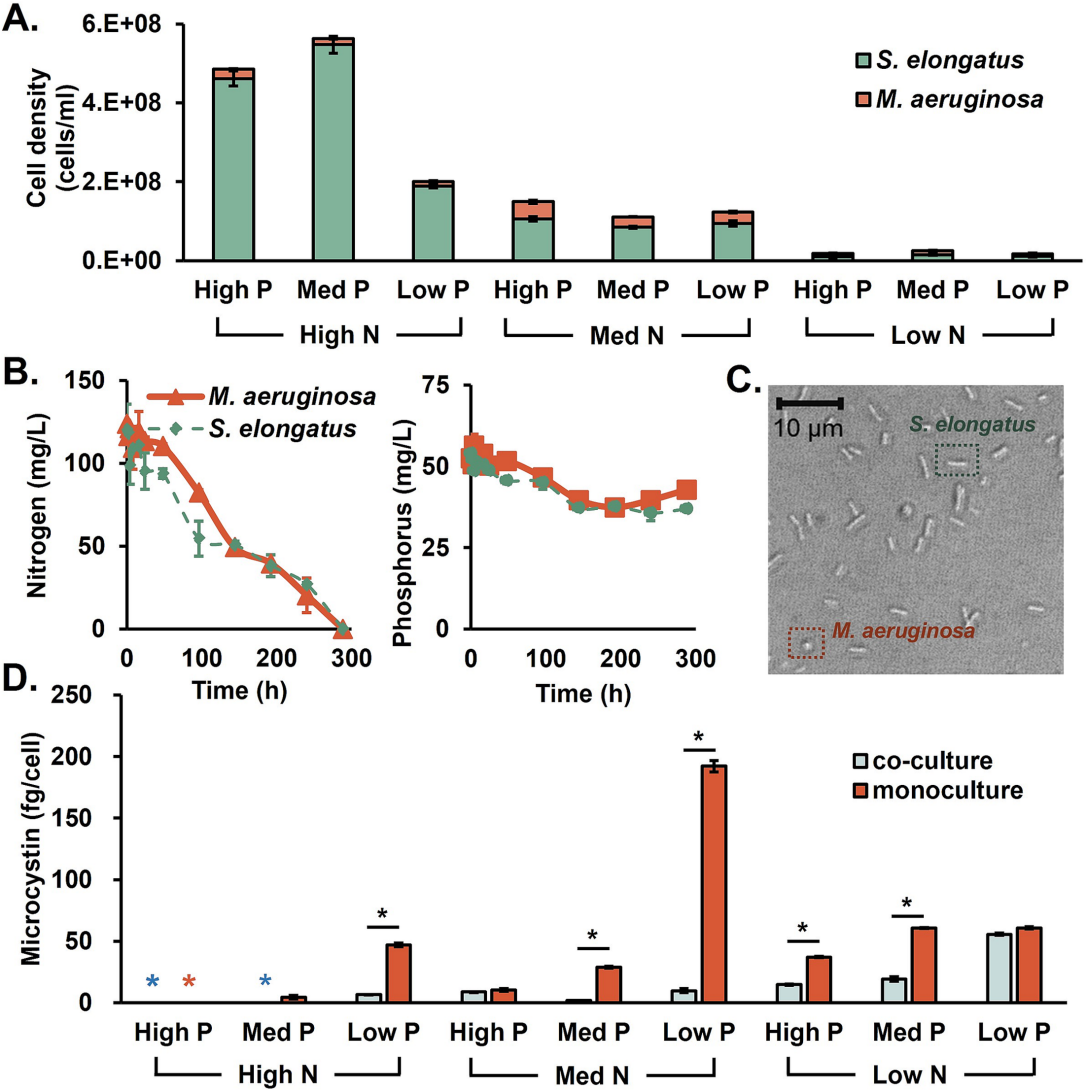
The characteristics of *M. aeruginosa* and *S. elongatus* cocultures. **(A)** The population of both species, **(B)** the N and P consumptions in monocultures, **(C)** a microscopic image of coculture, and **(D)** microcystin production by *M. aeruginosa* monoculture and coculture under various N and P conditions. Black asterisks indicate significant differences between the coculture and monoculture conditions (*t*-test, *p* < 0.05).

Knowing the microcystin production of *M. aeruginosa* monoculture, the biological competition with non-toxin-producing cyanobacteria, *S. elongatus*, was tested. Two species were grown together in a 1:1 ratio inoculation (2.5%:2.5% v/v). In *M. aeruginosa* monocultures, the total cell density could reach up to 3·10^8^ cells/mL in nutrient-rich conditions. Figure 2 shows the cell density of each species in the cocultured system. Cells were counted by taking microscopic images in the early stationary phase of day 14 (rod: *S. elongatus*; spheres: *M. aeruginosa*). Cocultures with *S. elongatus* limited *M. aeruginosa* growth by 92% in which the *S. elongatus* population was dominant in N- and P-sufficient conditions. The microcystin in cocultures was generally less than *M. aeruginosa* monocultures (Figure 2D).

### 3.3 Algicide effects on *Microcystis aeruginosa* microcystin production in monocultures and cocultures

**Figure 3:**
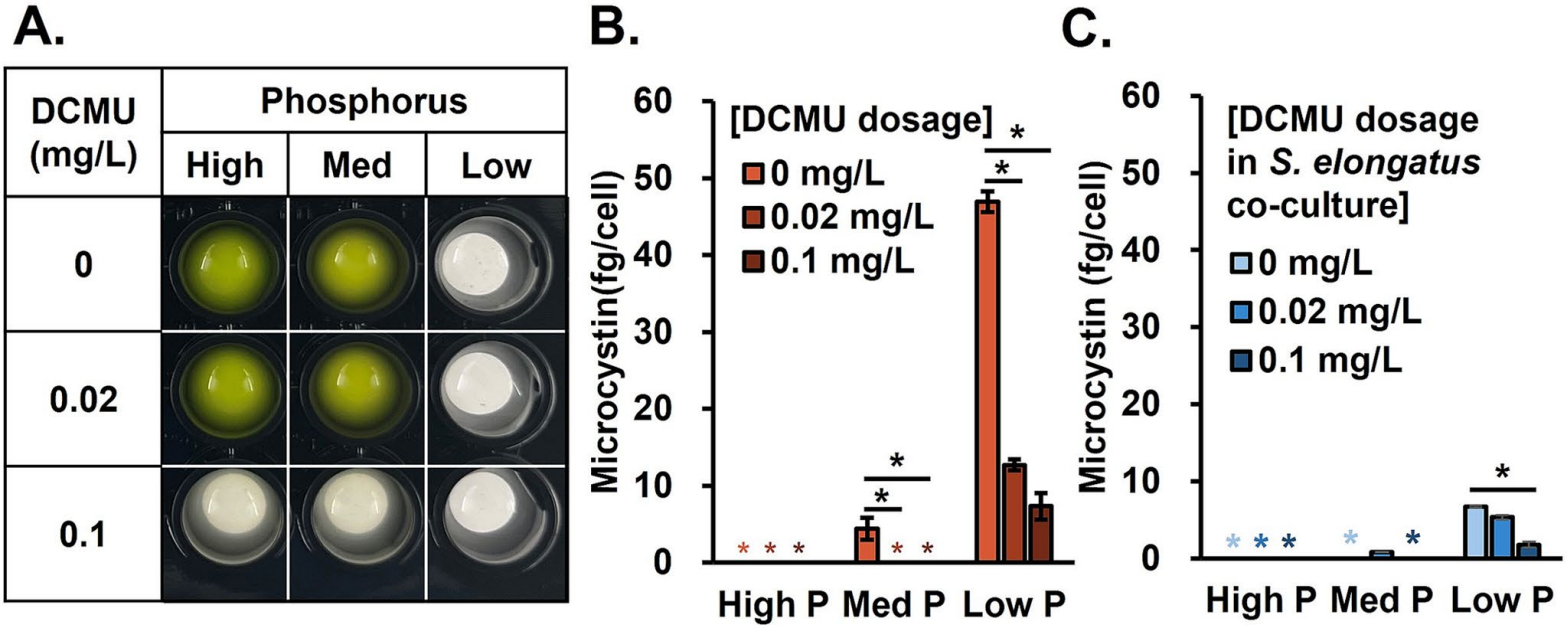
DCMU effect. **(A)** *Microcystis aeruginosa* monocultures, **(B)** microcystin production with high N and various P levels after DCMU treatments, and **(C)** microcystin production in a coculture system with *S. elongatus* under DCMU stress. Black asterisks indicate significant differences in microcystin production compared to the no DCMU dosage (ANOVA, *p* < 0.05).

The microcystin control by non-toxic cyanobacteria, *S. elongatus*, was confirmed. To further decrease the microcystin, an algicidal approach was brought. The primary mode of action is photosynthesis inhibition, which has been applied for herbicide development. Multiple studies have expanded the usage of herbicides to algicides to control HABs (Endo and Omasa, 2004; El-Sheekh et al., 1994; Kim et al., 2012). We performed the preliminary test of examining the performance of three different candidates (acetylacetone, copper sulfate, and DCMU) based on previous studies (Yilimulati et al., 2022, Murray-Gulde et al., 2002, Kirilovsky et al., 1994; Appendix H) and decided to use DCMU for our main study for two reasons: (1) DCMU effectively limited *M. aeruginosa* growth even at a very low concentration and (2) the tolerance of *S. elongatus* to DCMU (0.2 mg/L) was much higher than *M. aeruginosa*, which may allow the survival of *S. elongatus* in a 0.1 mg/L dosage while *M. aeruginosa* is fully inhibited. The following tests showed that 0.1 mg/L of DCMU could negatively impact *M. aeruginosa* growth in all test phosphorus concentrations (Figure 3A). DCMU dosage of 0.02 mg/L reduced the microcystin production by 75% under the low P condition (Figure 3B). We further analyzed microcystin in the *S. elongatus* cocultured system in the same DCMU dosage set to investigate whether there is a synergistic microcystin control (Figure 3C). As expected, nutrient availability influenced the growth performance (Appendix I). Under high N but low P conditions, the administration of DCMU at 0.1 mg/L level greatly reduced the microcystin production.

### 3.4 Microbial growth and microcystin production at lower temperatures

**Figure 4:**
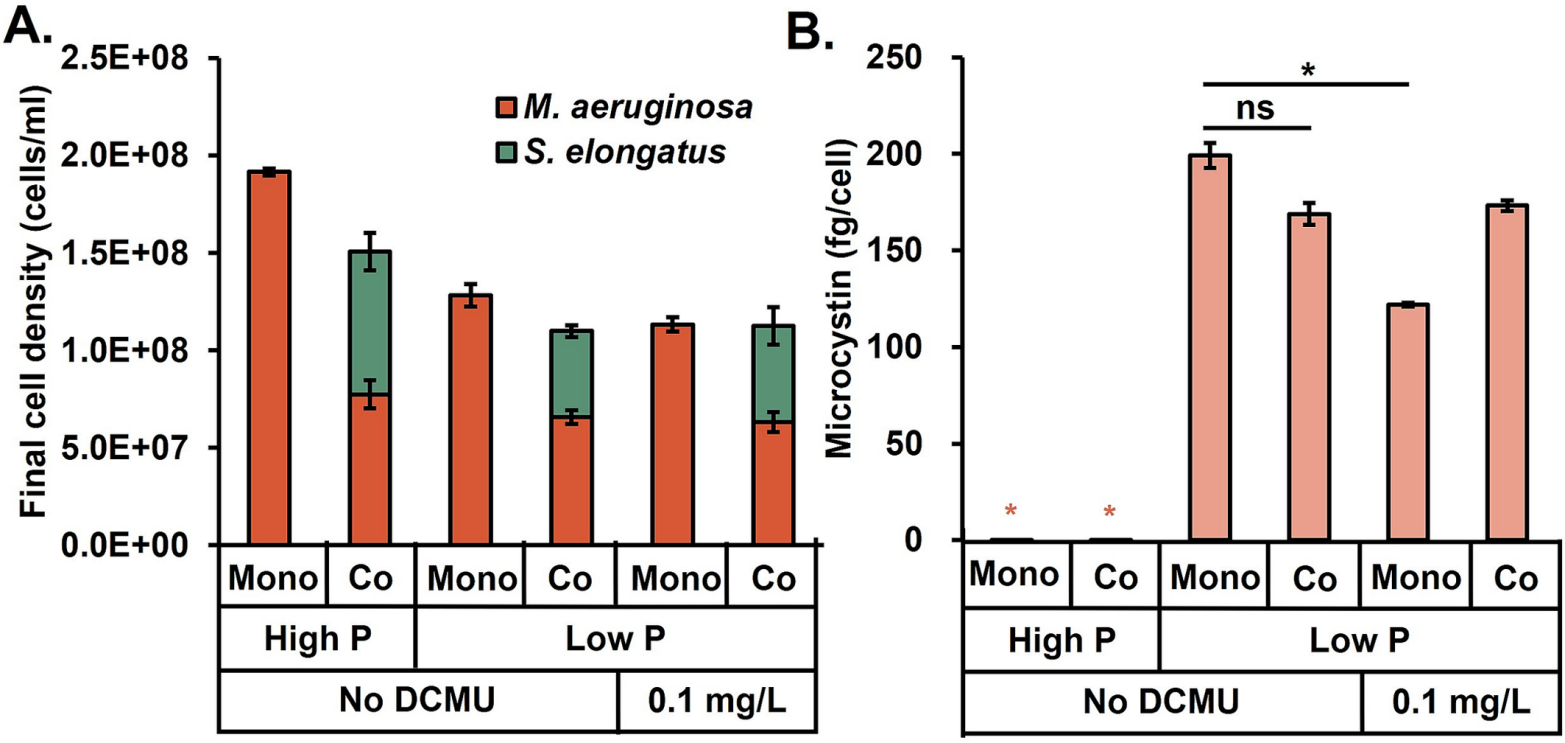
**(A)** *Microcystis aeruginosa* growth and **(B)** microcystin production at room temperature (23°C) under different combinations of cultivation conditions of nutrient limitation, cyanobacterial coculture, and DCMU treatment. Black asterisks indicate a significant difference between the two conditions; ns stands for no significant difference (*t*-test, *p* < 0.05). Abbreviations: mono, monoculture; co, coculture.

The above results were from algal cultivations under 30°C, which mimicked the hot summer weather. To investigate microcystin production at different temperatures, we repeated experiments at 23°C (Figure 4). For both monoculture and coculture, the microcystin was below the detection limit at high N and P conditions, while the microcystin concentration was highest in the low P condition (note: these observations were the same as the 30°C conditions). On the other hand, *S. elongatus* growth became much slower at room temperature, and the co-presence of *S. elongatus* did not significantly alleviate microcystin generation. Finally, the DCMU treatment moderately reduced the microcystin production in the monoculture system. However, the coculture could not further enhance the DCMU’s microcystin reduction at the low temperature.

### 3.5 Simulation of cyanobacterial growth and microcystin production

**Figure 5:**
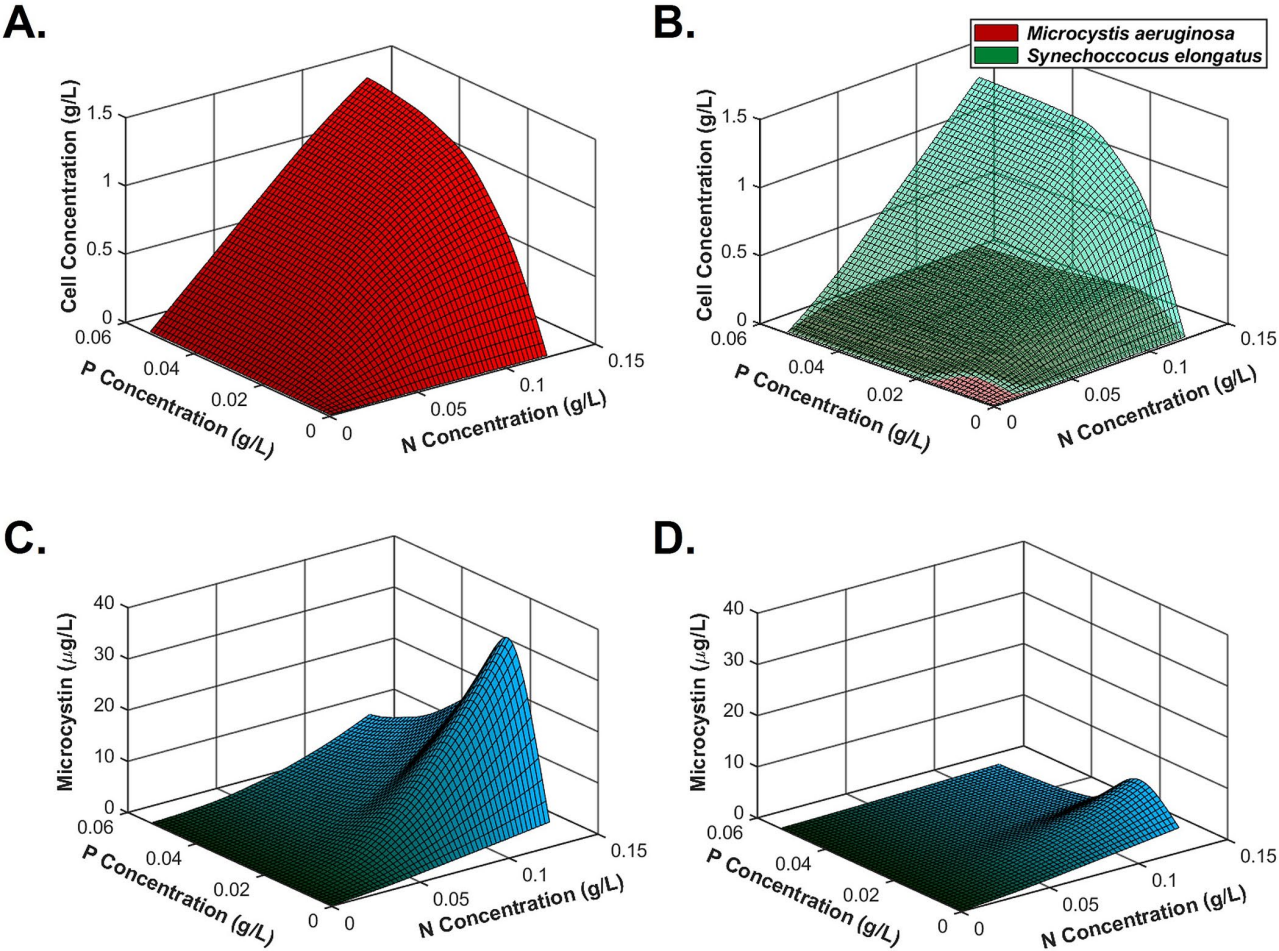
Simulation of biomass growth and microcystin production using a MATLAB-based kinetic model based on the experiment results. **(A)** *M. aeruginosa* monoculture growth, **(B)** coculture growth with *S. elongatus* under sufficient nutrients (green: *S. elongatus*; red: *M. aeruginosa*), **(C)** microcystin production in a monoculture system, and **(D)** microcystin production in the coculture system.

After testing all conditions, a kinetic model simulated algal monoculture and coculture growth (based on Equations 1, 2), toxin production (based on Equation 3), and nutrient consumption (based on Equations 4–6) under CO_2_-sufficient conditions. Figures 5A,B successfully simulated lower *M. aeruginosa* final cell density than *S. elongatus* in the coculture system, meaning that *M. aeruginosa* biomass was suppressed due to interactions between the two species. Figures 5C,D describe that microcystin production could be enhanced by high N and low P, while coculture conditions could reduce total microcystin concentrations compared to *M. aeruginosa* monoculture. Entire growth and nutrient consumption trends of monoculture and coculture are found in Appendix J. To solve the ordinary differential equations, all units were unified as mass per volume. Based on the trend, we can easily estimate the mass per cell unit of microcystin as well.

## 4 Discussion

This study highlights new perspectives on *M. aeruginosa*-driven cyanobacterial bloom control by utilizing non-toxic cyanobacteria. First, we conducted the control study to confirm the microcystin increase in P limitation. Cyanobacterial metabolic responses lead to upregulations of secondary metabolite production (Romano et al., 2015). The trend may be more active for cyanobacterial species that produce nitrogen-containing toxins to outcompete other species when the nutrients are limited in the environment (Brandenburg et al., 2020). The P-limitation stress induces microcystin-producing gene transcription (Pimentel and Giani, 2014) to promote peptide synthetase (*mcy*B) for microcystin synthesis (Figure 1). In addition, the increased light transmission resulting from less biomass production at a lower P concentration promotes N assimilation and high photosynthesis activities (Hellweger et al., 2022). Because of microcystin’s structure as a cyclic non-ribosomal peptide, accessibility to a high N concentration under low P conditions accelerates microcystin production. This finding demonstrates the importance of careful control of macronutrients (N and P) in remediating HABs.

Second, the co-existence of another cyanobacteria, *S. elongatus*, suppressed *M. aeruginosa* growth and toxin production. This competition became beneficial in limiting microcystin production under sufficient N and low P conditions, achieving an 85.9–94.9% reduction compared to *M. aeruginosa* monoculture by resource limitation and metabolic energy reallocation for (Baer et al., 2023; Gui et al., 2021). However, room temperature could not support the active growth of *S. elongatus* because the optimal temperature for the growth is 37–41°C (Yu et al., 2015). So far, no reports indicate that *S. elongatus* can produce specific microcystin degradation enzymes for direct microcystin control. As hypothesized, the indirect microcystin control by nutrient competition is the most feasible mechanism, as model simulations indicate (Figure 5). This competition can generally limit *M. aeruginosa* growth and toxin synthesis as long as the growth of *S. elongatus* is much higher than that of *M. aeruginosa*. It is also possible that there might be some unknown metabolites secreted by the fast-growing cyanobacteria that antagonistically inhibit *M. aeruginosa* growth, which requires further research efforts to elucidate. Since synthetic biology tools for *S. elongatus* are available, it is possible to engineer kill switches in *S. elongatus* so they can be removed from the environment after outcompeting toxin-producing *M. aeruginosa* (Rottinghaus et al., 2022). In another scenario, the over-growing *S. elongatus* biomass in smaller waterbodies can successfully dominate the population and be recovered and utilized as feedstocks for biomanufacturing or animal feed (Lin et al., 2020; Song et al., 2016).

Third, DCMU herbicide effectively controlled *M. aeruginosa* growth and microcystin production (as low as 0.02 mg/L) as the algicide. Specifically, the DCMU treatment blocked the electron transfer at the quinone-B site of photosystem II and limited the production of NADPH, which also suppressed the transcription of the microcystin-producing genes (Sevilla et al., 2012). In addition, DCMU is often present in surface and groundwater due to herbicide leaching from croplands, which can affect intracellular detoxification (Dragone et al., 2015). The results of this study indicated that algicidal treatment in low P conditions could be a helpful strategy to control HABs, while the presence of growth competition from other microalgae species (such as DCMU-tolerant *S. elongatus*) in algal consortia could further suppress *M. aeruginosa* toxin synthesis. However, the herbicidal treatment may affect other phytoplankton species. More detailed and cautious dosage determination and algal community tests should be a potential future study.

## 5 Conclusion

Representative cyanobacterial bloom-forming *M. aeruginosa* was studied to understand its behaviors in the presence of non-toxic *S. elongatus*. We found that a high N concentration with P limitation increased microcystin production by *M. aeruginosa*, which was suppressed by adding *S. elongatus*. *M. aeruginosa* population and microcystin were controlled. The additional application of algicidal DCMU reduced microcystin production further in the cocultured system. Collectively, our findings demonstrate that benign cyanobacterial species can be potentially used as a non-parasitic candidate to outcompete toxin-producing microalgae and produce biomass (as valuable feedstock) from CO_2_.

## Data Availability

The raw data supporting the conclusions of this article will be available by the authors, without undue reservation.
