# Erratum: The use of a benign fast-growing cyanobacterial species to control microcystin synthesis from *Microcystis aeruginosa*

**DOI:** 10.3389/fmicb.2025.1557505

**Published:** 2025-01-23

**Authors:** Frontiers Production Office

**Affiliations:** Frontiers Media SA, Lausanne, Switzerland

**Keywords:** coculture, DCMU, harmful algal blooms, *Microcystis aeruginosa*, *Synechococcus elongatus*

Due to a production error, the abstract of this article contained numerous inaccuracies. The corrected abstract appears below:

**Introduction:** *Microcystis aeruginosa* (*M. aeruginosa*), one of the most abundant blue-green algae in aquatic environments, produces microcystin by causing harmful algal blooms (HABs). This study investigated the combined effects of nutrients and competition among cyanobacterial subpopulations on the synthesis of microcystin-LR.

**Methods:** Under varying nitrogen and phosphorus concentrations, cyanobacterial coculture, and the presence of algicidal DCMU, the growth was monitored by optical density analysis or microscopic counting, and the microcystin production was analyzed using high-performance liquid chromatography-UV. Furthermore, growth and toxin production were predicted using a kinetic model.

**Results and discussion:** First, coculture with the fast-growing cyanobacterium *Synechococcus elongatus* UTEX 2973 (*S. elongatus*) reduced *M. aeruginosa* biomass and microcystin production at 30°C. Under high nitrogen and low phosphorus conditions, *S. elongatus* was most effective, limiting *M. aeruginosa* growth and toxin synthesis by up to 94.7% and 92.4%, respectively. Second, this biological strategy became less effective at 23°C, where *S. elongatus* grew more slowly. Third, the photosynthesis inhibitor DCMU (3-(3,4-dichlorophenyl)-1,1-dimethylurea) inhibited *M. aeruginosa* growth (at 0.1 mg/L) and microcystin production (at 0.02 mg/L). DCMU was also effective in controlling microcystin production in *S. elongatus*–*M. aeruginosa* cocultures. Based on the experimental results, a multi-substrate, multi-species kinetic model was built to describe coculture growth and population interactions.

**Conclusion:** Microcystin from representative toxin-producing *M. aeruginosa* can be controlled by coculturing fast-growing benign cyanobacteria, which can be made even more efficient if appropriate algicide is applied. This study improved the understanding of the biological control of microcystin production under complex environmental conditions.

A correction has been made to Section 2 “Materials and methods”, Section 2.6 “Model development and simulations”, paragraph one. The value “n” has been formatted as superscript:

“An empirical term α = β (N/P)^n^ describes the toxin accumulation as a function of N:P ratios:”

A correction has been made to Section 3 “Results,” Section 3.1 “Effects of N and P on Microcystis aeruginosa monoculture growth and microcystin production,” paragraph one. The word “production” has been un-bolded.

A correction has been made to Section 3 “Results,” Section 3.5, “Simulation of cyanobacterial growth and microcystin production,” paragraph one. The order of the cited equations in the following sentence has been corrected:

“After testing all conditions, a kinetic model simulated algal monoculture and coculture growth (based on Equations 1, 2), toxin production (based on Equation 3), and nutrient consumption (based on Equations 4–6) under CO_2_-sufficient conditions.”

The publisher apologizes for these mistakes.

The original article has been updated.

