# Supplementary material for: The use of a benign fast-growing cyanobacterial species to control microcystin synthesis from Microcystis aeruginosa
Source: Front Microbiol. 2024 Dec 5;15:1461119. doi: 10.3389/fmicb.2024.1461119 (PMC11655507; doi:10.3389/fmicb.2024.1461119)
Supplement: Supplementary file 1 [file Supplementary_file_1.docx]

# Supporting Information

**The use of a benign fast-growing cyanobacterial species to control microcystin synthesis from *Microcystis aeruginosa***

Hakyung Lee^1^, Vincent Xu^1^, Jinjin Diao^1^, Runyu Zhao^1^, Moshan Chen^1^, Tae Seok Moon^1^, Haijun Liu^2^, Kimberly M. Parker^1^, Young-Shin Jun*^,1^, and Yinjie J. Tang*^,1^

^1^ Department of Energy, Environmental and Chemical Engineering, Washington University in St. Louis, St. Louis, Missouri 63130, USA

^2^ Department of Biology, Saint Louis University, St. Louis, Missouri 63108, USA

# Appendix A. The composition of (1) Bold 3 N medium and (2) BG 11 medium

## A.1.1. Bold 3N medium composition (1)

| Component | Concentration |
| --- | --- |
| NaNO_3_ | 8.82 mM |
| CaCl_2_•2H_2_O | 0.17 mM |
| MgSO_4•_7H_2_O | 0.3 mM |
| K_2_HPO_4_ | 0.43 mM |
| KH_2_PO_4_ | 1.29 mM |
| NaCl | 0.43 mM |
| P-IV Metal solution | 0.6 % |
| Soilwater: GR+ Medium | 4 % |
| Vitamin B_12_ | 0.1 % |

## A.1.2. P-IV Metal solution composition

| Component | Concentration |
| --- | --- |
| Na_2_EDTA•2H_2_O | 2 mM |
| FeCl_3_•6H_2_O | 0.36 mM |
| MnCl_2_•4H_2_O | 0.21 mM |
| ZnCl_2_ | 0.037 mM |
| CoCl_2_•6H_2_O | 0.0084 mM |
| Na_2_MoO_4_•2H_2_O | 0.017 mM |

## A.2.1. BG-11 medium composition (2)

| Component | Concentration |
| --- | --- |
| NaNO_3_ | 17.6 mM |
| K_2_HPO_4_ | 0.23 mM |
| MgSO_4_•7H_2_O | 0.3 mM |
| CaCl_2_•2H_2_O | 0.24 mM |
| Citric Acid•H_2_O | 0.031 mM |
| Ferric Ammonium Citrate | 0.021 mM |
| Na_2_EDTA•2H_2_O | 0.0027 mM |
| Na_2_CO_3_ | 0.19 mM |
| BG-11 Trace Metals solution | 0.1 % |

## A.2.2. BG-11 Trace metal solution composition

| Component | Concentration |
| --- | --- |
| H_3_BO_3_ | 46 mM |
| MnCl_2_•4H_2_O | 9 mM |
| ZnSO_4_•7H_2_O | 0.77 mM |
| Na_2_MoO_4_•2H_2_O | 1.6 mM |
| CuSO_4_•5H_2_O | 0.3 mM |
| Co(NO_3_)_2_•6H_2_O | 0.17 mM |

# Appendix B. Microcystin-LR sample extraction method

We collected a 5 ml sample of well-mixed algal culture, centrifuged it at 5000 rcf for 10 min at 4°C, then transferred the supernatant to another test tube. The precipitated cells were resuspended in deionized water, well mixed by vortexing, stored in packed ice for at least 5 min, and lysed by ultrasonication for 1 min, with a 5 s pause every 15 s (Q700 Sonicator, Qsonica). To remove particles, the sonicated samples and supernatant were filtered using a 0.45 µm syringe filter. Next, the filtrate was diluted by 10-fold. Each sample was extracted using the solid phase extraction (SPE) method using Supelco™ Visiprep™ SPE vacuum manifold from MilliporeSigma™ connected to the vacuum nozzle. Oasis^®^ HLB (Hydrophilic-Lipophilic-Balanced) cartridge was installed at the top of the manifold. First, the cartridge was conditioned using 3 ml of 100 % methanol at 20 kPa pressure. After that, the cartridge was equilibrated using 6 ml of Milli-Q water at 20 kPa. Then, 50 ml of prepared water sample passed the cartridge at sample pressure. Then, the cartridge was rinsed with 5 ml of 20 % methanol in water at 15 kPa. The rinsed cartridge was dried for 2 min. The wide-mouth glass tube was prepared inside the manifold. For the final extraction of the analyte, 5 ml of 100 % methanol passed the cartridge at 15 kPa. This extracted sample was dried inside the fume hood under gentle air flow for 24 hours. A completely dried glass tube was filled with 0.5 ml of 100 % methanol and vigorously mixed for 10 min after sealing it using parafilm. Then, the sample was transported to the HPLC vial for analysis (3, 4).

# Appendix C. RT-qPCR primer information for microcystin-producing gene quantification

| Target gene | Sequences (5`-3`) | References |
| --- | --- | --- |
| 16S rRNA  in *M. aeruginosa* | Forward: GTAGCAGGAATTCCCAGTGTAG  Reverse: TTCGTCCCTGAGTGTCAGATA | (5) |
| *mcy*B | Forward: TGGGAAGATGTTCTTCAGGTATCCAA  Reverse: AGAGTGGAAACAATATGATAAGCTAC | (6) |
| *mcy*D | Forward: GGTTCGCCTGGTCAAAGTAA  Reverse: CCTCGCTAAAGAAGGGTTGA | (7) |

# Appendix D. Parameters for prediction model

| Parameter | Description | Value | Unit | References |
| --- | --- | --- | --- | --- |
| T | Temperature | 30 | °C | Experiment  condition |
| V | Culture Volume | 0.05 | L | Experiment  condition |
| I | Incident Light Intensity | 200 | µmol·photons·m^2^·s^-1^ | Experiment  condition |
| A | Light Absorption Coefficient | 14.7 | L·g^-1^ | (8) |
| S_CO2_* | CO_2_ Baseline Concentration | 0.0005 | g·L^-1^ | Based on Henry’s law |
| µ_max,A_ | Max Growth Rate_A | 0.05 | h^-1^ | Assumed |
| µ_max,B_ | Max Growth Rate_B | 0.1 | h^-1^ | Assumed |
| k_N,A_ | N Half Saturation Constant_A | 0.016 | g·L^-1^ | Adjusted considering N uptake rate |
| k_N,B_ | N Half Saturation Constant_B | 0.032 | g·L^-1^ | (9) |
| k_P,A_ | P Half Saturation Constant_A | 0.00525 | g·L^-1^ | Adjusted considering P uptake rate |
| k_P,B_ | P Half Saturation Constant_B | 0.0105 | g·L^-1^ | (9) |
| k_C,A_ | CO_2_ Half Saturation Constant_A | 0.00026 | g·L^-1^ | (10) |
| k_C,B_ | CO_2_ Half Saturation Constant_B | 0.00026 | g·L^-1^ | (10) |
| k_L,A_ | Light Half Saturation Constant_A | 14 | µmol·photons·m^2^·s^-1^ | (8) |
| k_L,B_ | Light Half Saturation Constant_B | 14 | µmol·photons·m^2^·s^-1^ | (8) |
| k_d,A_ | Death Constant_A | 0.00005 | h^-1^ | Assumed |
| k_d,B_ | Death Constant_B | 0.00005 | h^-1^ | Assumed |
| k_La1_ | CO_2_ Mass Transfer Coefficient | 70 | h^-1^ | Assumed |
| Y_X/N,A_ | N Biomass Yield_A | 0.081 | gN·g biomass^-1^ | (11) |
| Y_X/N,B_ | N Biomass Yield_B | 0.081 | gN·g biomass^-1^ | (11) |
| Y_X/P,A_ | P Biomass Yield_A | 0.0087 | gP·g biomass^-1^ | Assumed |
| Y_X/P,B_ | P Biomass Yield_B | 0.0087 | gP·g biomass^-1^ | Assumed |
| Y_X/C,A_ | CO2 Biomass Yield_A | 1.8 | g CO2/g biomass | (10) |
| Y_X/C,B_ | CO2 Biomass Yield_B | 1.8 | g CO2/g biomass | (10) |
| Y_T_ | Maximum Toxin Cell Mass Fraction (without chemical inhibition) | 5·10^-6^ | g toxin  /g A biomass | Adjusted considering experiment data |
| Y_T_ | Maximum Toxin Cell Mass Fraction (with chemical inhibition) | 4·10^-4^ | g toxin  /g A biomass | Adjusted considering experiment data |

**Note: some parameters in this model are assumed and may not be strictly accurate; however, they are within a reasonable range for describing the growth phenomena of the algal species of interest.**

# Appendix E. MATLAB code for the prediction model

The MATLAB code to generate a prediction model is provided below. The detailed data and individual code generation is available on GitHub:
https://github.com/xav1002/microcystin_reduction.

## E.1. Batch function code

**
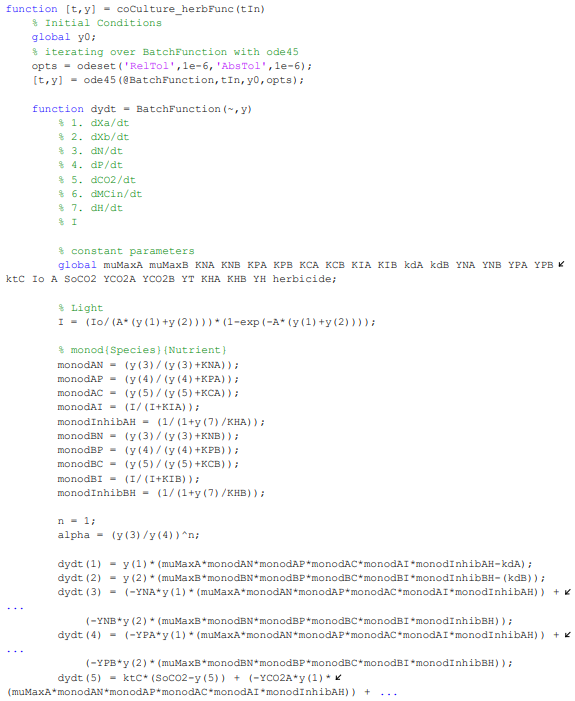


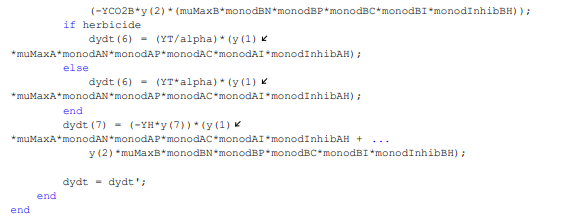
**

## E.2. Main MATLAB code for loading data, function, and creating plots


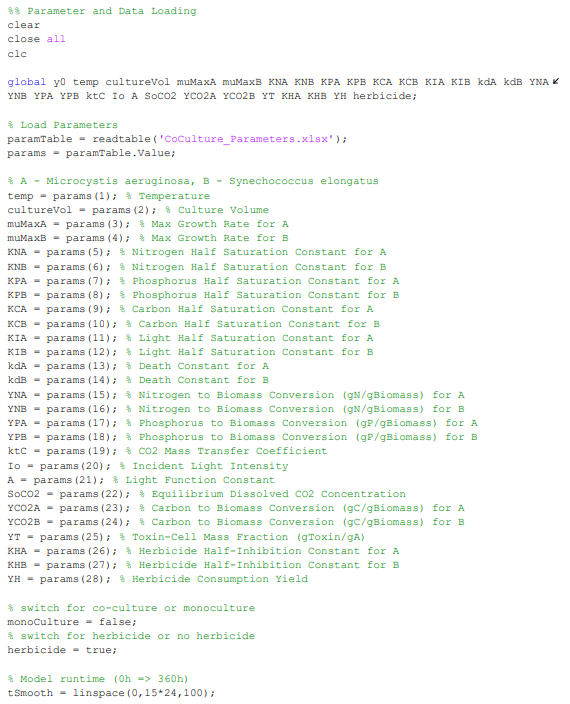


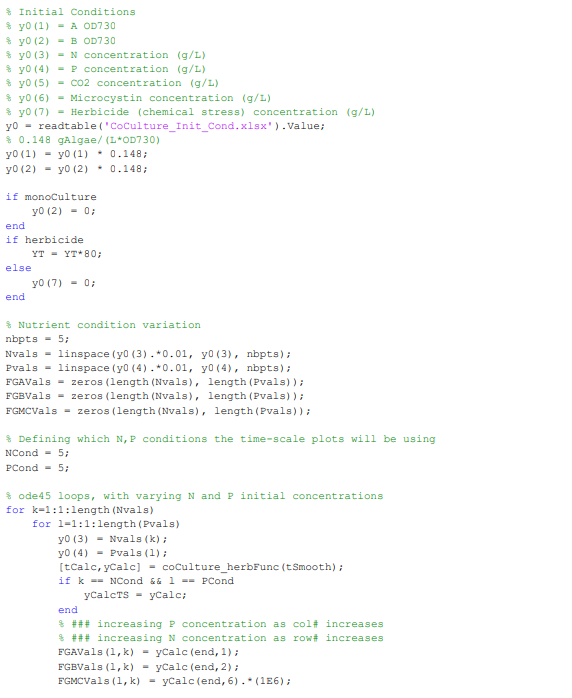


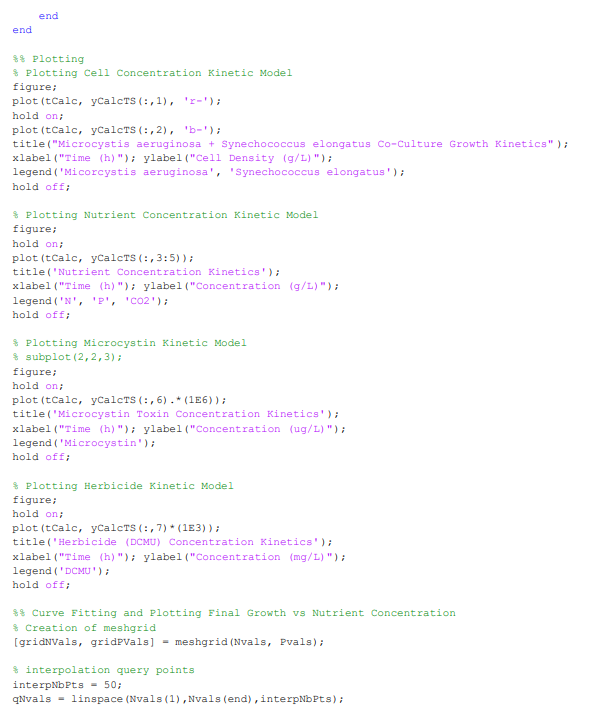


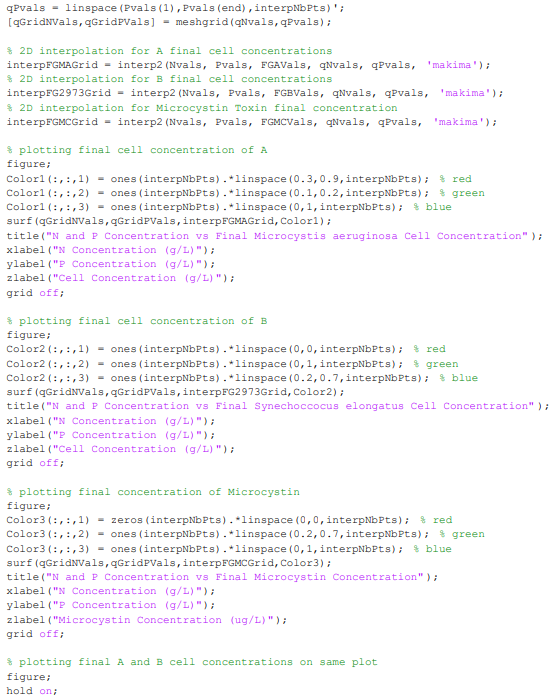


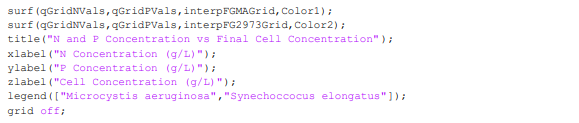


# Appendix F. Cell density during *Microcystis aeruginosa* growth in various nitrogen and phosphorus concentrations (n=3). The correlation between nitrogen concentrations and cell density in day 14 has significant positive correlation with Pearson coefficient of 0.78 (*p* < 0.01)

| Phosphorus | Nitrogen | Day 8  cell density (± SE) (cell/ml) | Day 14  cell density (± SE) (cell/ml) |
| --- | --- | --- | --- |
| High | High | 1.4E+08 (± 6.6E+06) | 5.4E+08 (± 6.9E+06) |
|  | Med | 1.0E+08 (± 6.1E+06) | 1.4E+08 (± 6.0E+06) |
|  | Low | 0.8E+08 (± 6.1E+06) | 1.0E+08 (± 6.1E+06) |
| Med | High | 1.6E+08 (± 6.2E+06) | 5.6E+08 (± 9.8E+06) |
|  | Med | 1.0E+08 (± 6.2E+06) | 1.2E+08 (± 6.0E+06) |
|  | Low | 0.8E+08 (± 6.1E+06) | 1.0E+08 (± 6.0E+06) |
| Low | High | 1.0E+08 (± 6.3E+06) | 1.4E+08 (± 6.1E+06) |
|  | Med | 1.0E+08 (± 6.1E+06) | 1.4E+08 (± 6.1E+06) |
|  | Low | 0.8E+07 (± 6.1E+06) | 1.0E+08 (± 6.1E+06) |

# Appendix G. Microcystin production in *M. aeruginosa* monoculture in day 4, day 8, day 10, and day 14


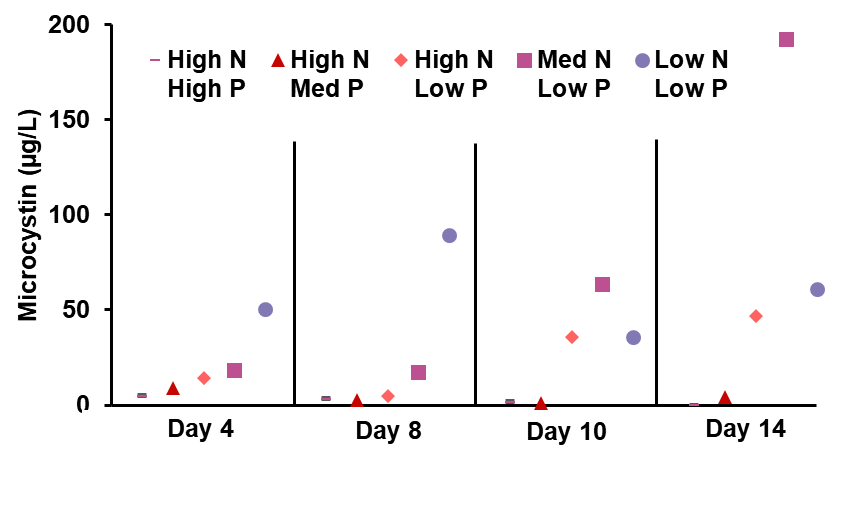


# Appendix H. Herbicide effects on *Synechococcus elongatus* monoculture (A, B, C) and *Microcystis aeruginosa* monoculture (D, E, F) growth under herbicide treatments

**
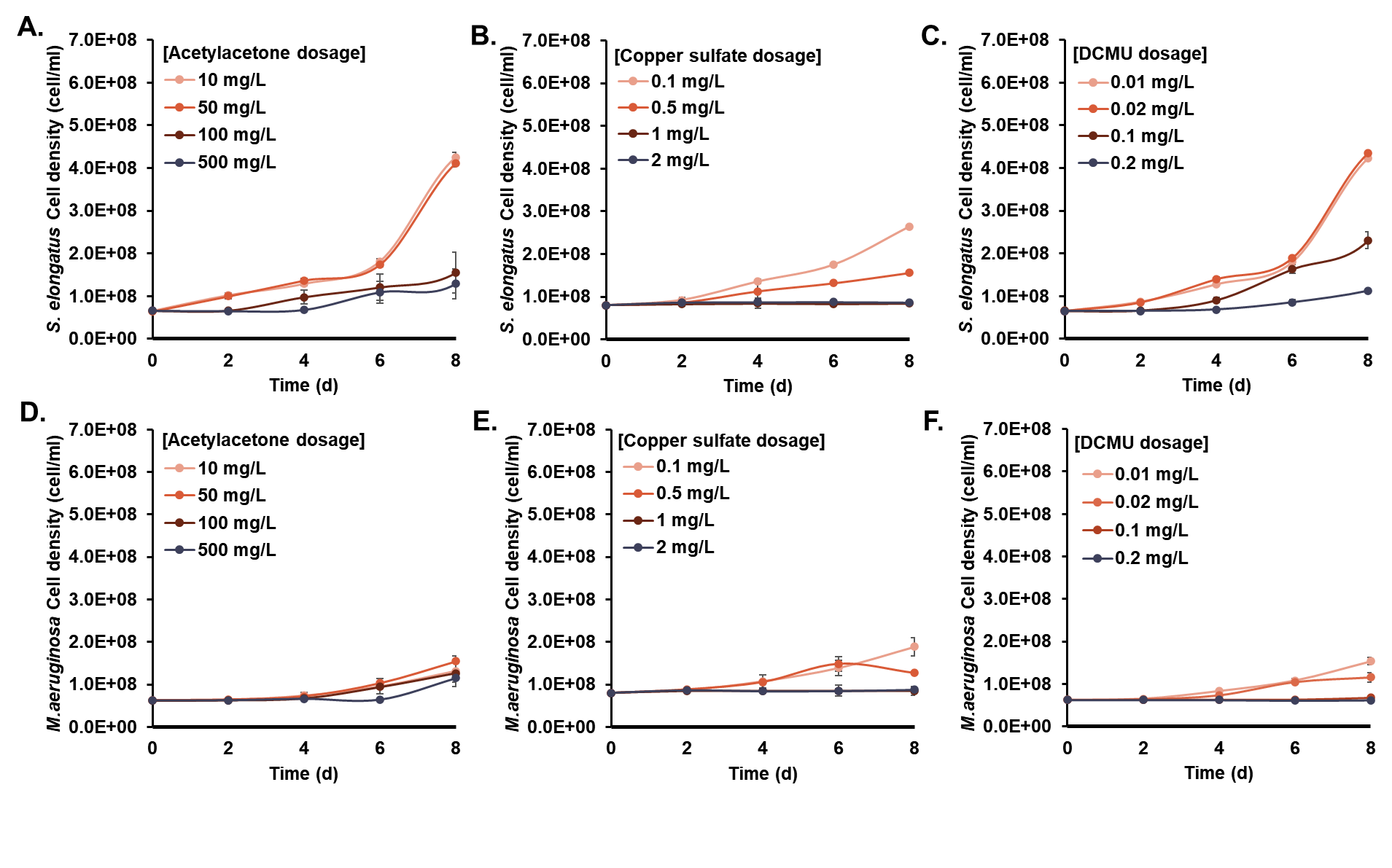
**

# Appendix I. Cell density during *Microcystis aeruginosa* and *Synechococcus elongatus* growth in the coculture system under various nitrogen and phosphorus concentrations with the 0.1 mg/L DCMU treatment (n=3)

| Phosphorus | Nitrogen | Exponential phase  cell density (± SE) (cell/ml) | Stationary phase  cell density (± SE) (cell/ml) |
| --- | --- | --- | --- |
| High | High | 1.5E+08 (± 9.2E+06) | 3.4E+08 (± 3.5E+07) |
|  | Med | 1.2E+08 (± 2.5E+06) | 1.2E+08 (± 2.5E+06) |
|  | Low | 9.7E+07 (± 1.3E+07) | 7.3E+07 (± 1.1E+05) |
| Med | High | 1.8E+08 (± 9.5E+06) | 4.3E+08 (± 1.1E+07) |
|  | Med | 1.2E+08 (± 4.8E+06) | 1.1E+08 (± 5.3E+06) |
|  | Low | 8.2E+07 (± 3.6E+05) | 7.4E+07 (± 6.0E+04) |
| Low | High | 1.4E+08 (± 1.5E+06) | 1.7E+08 (± 1.9E+06) |
|  | Med | 1.2E+08 (± 9.6E+06) | 1.1E+08 (± 3.5E+06) |
|  | Low | 8.3E+07 (± 2.8E+05) | 7.3E+07 (± 2.0E+05) |

# Appendix J. Simulation of biomass growth and microcystin production using MATLAB based kinetic model based on the experiment results. (A) *M. aeruginosa* growth under high N and P nutrients, (B) *M. aeruginosa* and *S. elongatus* biomass growth under sufficient nutrients (green: *S. elongatus*; red: *M. aeruginosa*), and (C) Nutrient consumption kinetics in the monoculture system under high N and P conditions, (D) Nutrient consumption kinetics in the coculture system under high N and P conditions


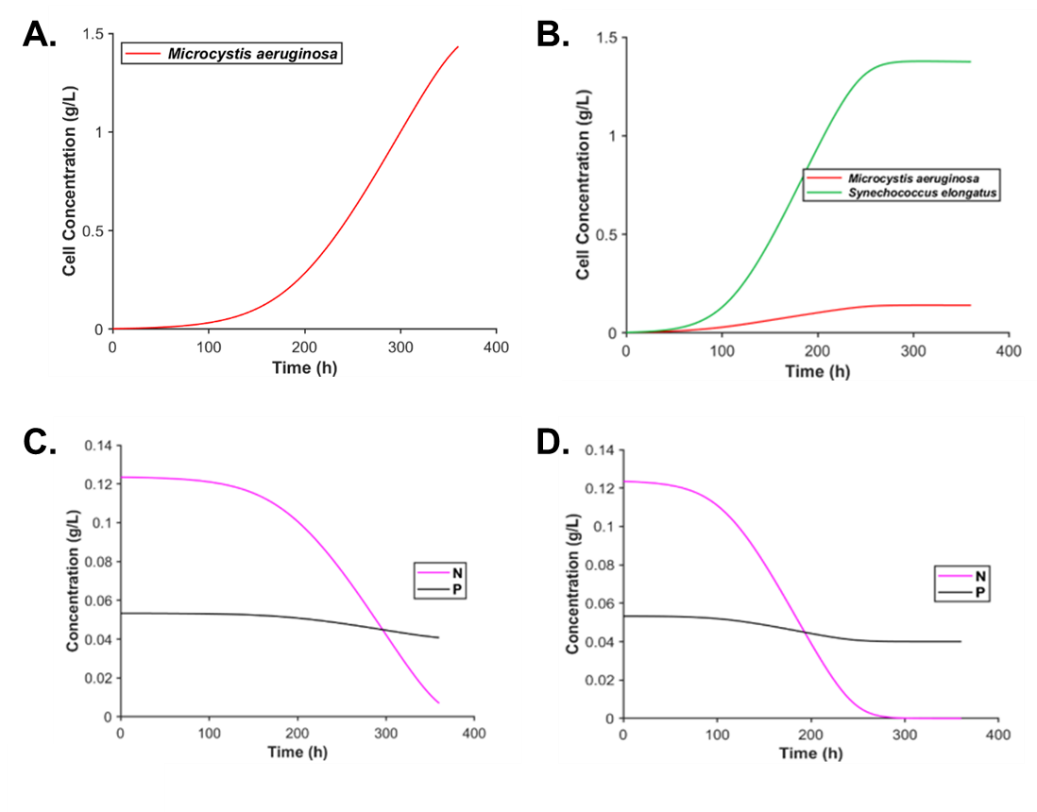


# References

1. The University of Texas at Austin U. Bold 3N Medium Recipe 2018 [Available from: <https://web.biosci.utexas.edu/utex/Media%20PDF/bold-3n-medium.pdf>.

2. The University of Texas at Austin U. BG-11 Medium Recipe 2018 [Available from: <https://web.biosci.utexas.edu/utex/Media%20PDF/bg-11-medium.pdf>.

3. Shoemaker J, Dan Tettenhorst, and A. Delacruz. Method 544. Determination of Microcystins and Nodularin in Drinking Water by Solid Phase Extraction and Liquid Chromatography/Tandem Mass Spectrometry (LC/MS/MS). In: Agency USEP, editor. Washington, DC2015.

4. Zervou S-K, Christophoridis C, Kaloudis T, Triantis TM, Hiskia A. New SPE-LC-MS/MS method for simultaneous determination of multi-class cyanobacterial and algal toxins. Journal of Hazardous Materials. 2017;323:56-66.

5. Perez JL, Chu T. Effect of zinc on Microcystis aeruginosa UTEX LB 2385 and its toxin production. Toxins. 2020;12(2):92.

6. Nonneman D, Zimba P. A Pcr-Based Test to Assess the Potential for Microcystin Occurrence in Channel Catfish Production Ponds 1,2. Journal of Phycology. 2002;38:230-3.

7. Kaebernick M, Neilan BA, Börner T, Dittmann E. Light and the transcriptional response of the microcystin biosynthesis gene cluster. Appl Environ Microbiol. 2000;66(8):3387-92.

8. Martínez ME, Camacho F, Jiménez JM, Espínola JB. Influence of light intensity on the kinetic and yield parameters of Chlorella pyrenoidosa mixotrophic growth. Process Biochemistry. 1997;32(2):93-8.

9. Aslan S, Kapdan IK. Batch kinetics of nitrogen and phosphorus removal from synthetic wastewater by algae. Ecological Engineering. 2006;28(1):64-70.

10. Spijkerman E, de Castro F, Gaedke U. Independent Colimitation for Carbon Dioxide and Inorganic Phosphorus. PLOS ONE. 2011;6(12):e28219.

11. Kumar K, Dasgupta CN, Das D. Cell growth kinetics of Chlorella sorokiniana and nutritional values of its biomass. Bioresource Technology. 2014;167:358-66.
